# Supplementary material for: Ancestry-specific polygenic risk scores are risk enhancers for clinical cardiovascular disease assessments
Source: Nat Commun. 2023 Nov 4;14:7105. doi: 10.1038/s41467-023-42897-w (PMC10625612; doi:10.1038/s41467-023-42897-w)
Supplement: Supplementary file 1 — Supplementary Information [file 41467_2023_42897_MOESM1_ESM.pdf]

Supplementary Material: Ancestry Specific Polygenic Risk Scores are Risk Enhancers for 10 year Cardiovascular Disease Risk Assessments

George B. Busby[1,*], Scott Kulm[1], Alessandro Bolli[1], Jen Kintzle[1], Paolo Di Domenico[1], Giordano Bottà[1,*], George B. Busby[1,*], Scott Kulm[1], Alessandro Bolli[1], Jen Kintzle[1], Paolo Di Domenico[1], and Giordano Bottà[1,*]

[1]Allelica Inc, 447 Broadway, New York, 10013, USA

[*]*Correspondence to* george@allelica.com *and* giordano@allelica.com

Supplementary Figures

Supplementary Figure 1: The methodological development of multiancestry Polygenic Risk Scores. We began with data from two prospective cohorts, the UK Biobank (UKBB) and the Multiethnic Study of Atherosclerosis (MESA). Details of the Genome Wide Association Studies (GWAS) used in this study are in Table Table 1. Breifly, GWAS were performed on subsets of individuals in the UKBB of African, European and South Asian genetic ancestries. We also obtained GWAS summary statistics from external sources (CARDIoGRAMplusC4D, Japanese 160k and Japanese 52k). We used Allelica's DISCOVER tool to process and combine these summary statistics with a version of the PRS-CSx tool optimised to run more quickly, generating 152 PRS panels. Allelica's PREDICT software was used to generate individual level PRS values for the remaining individuals from the MESA and UKBB cohorts who had not previously been utilized for GWAS. Within each ancestry, we identified the PRS with the greatest Odds Ratio per Standard Deviation in a Validation dataset comprising half the data and tested these in an independent Testing dataset comprising the other half of the individuals.

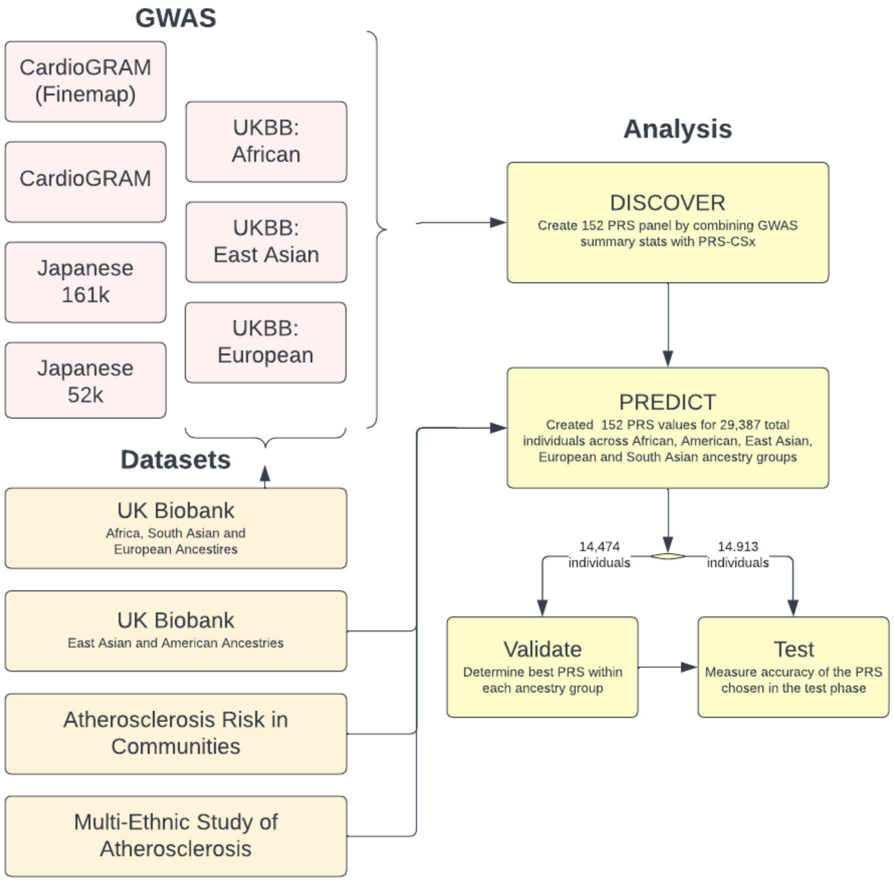

Supplementary Figure 2: Flow Chart Describing How the Testing and Validation Datasets were Created. The flow chart starts with an example ancestry group, and by asking questions regarding the cases in the ancestry group we determine how many cases and what cohorts should be placed into the validation and testing datasets. Within the middle of the flow chart we make explicit the ancestry groups that fall out of each questions. Lastly, during the analysis of the validation dataset we use the question in the flow chart to determine whether a meta PRS or bootstrapping based PRS should be employed to the testing dataset.

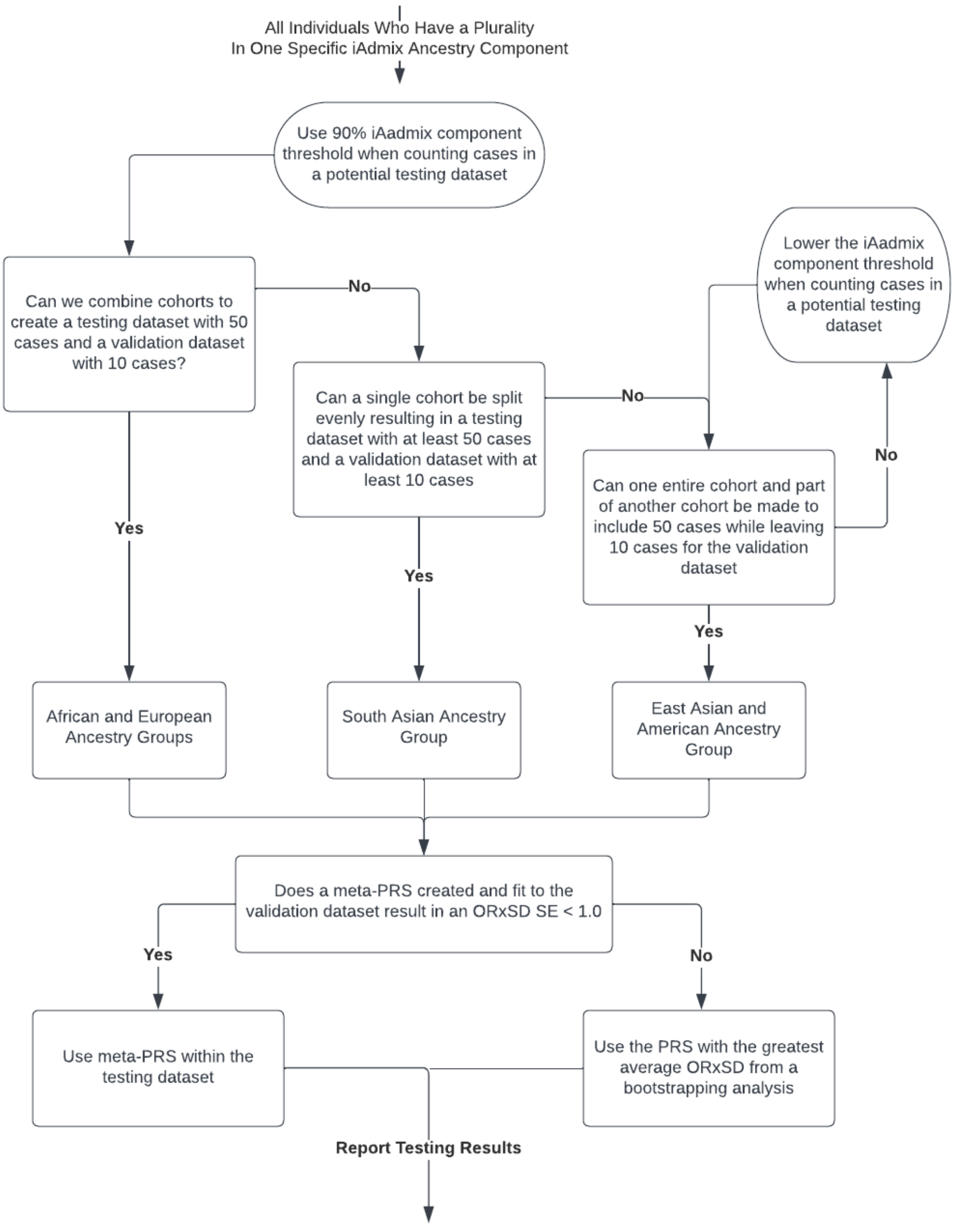

Supplementary Figure 3: Benchmarking of the Allelica_CAD_Multi_vJ PRS scores with three published scores. For each genetic ancestry group, we show the point estimate and 95% CI of the Area Under the Curve (AUC) for a range of logistic regression models (rows) adjusted for different covariates. The covariates included in the model are reported to the right of each panel row. Numbers of individuals in each genetic ancestry group are detailed in Supplementary Table 15.

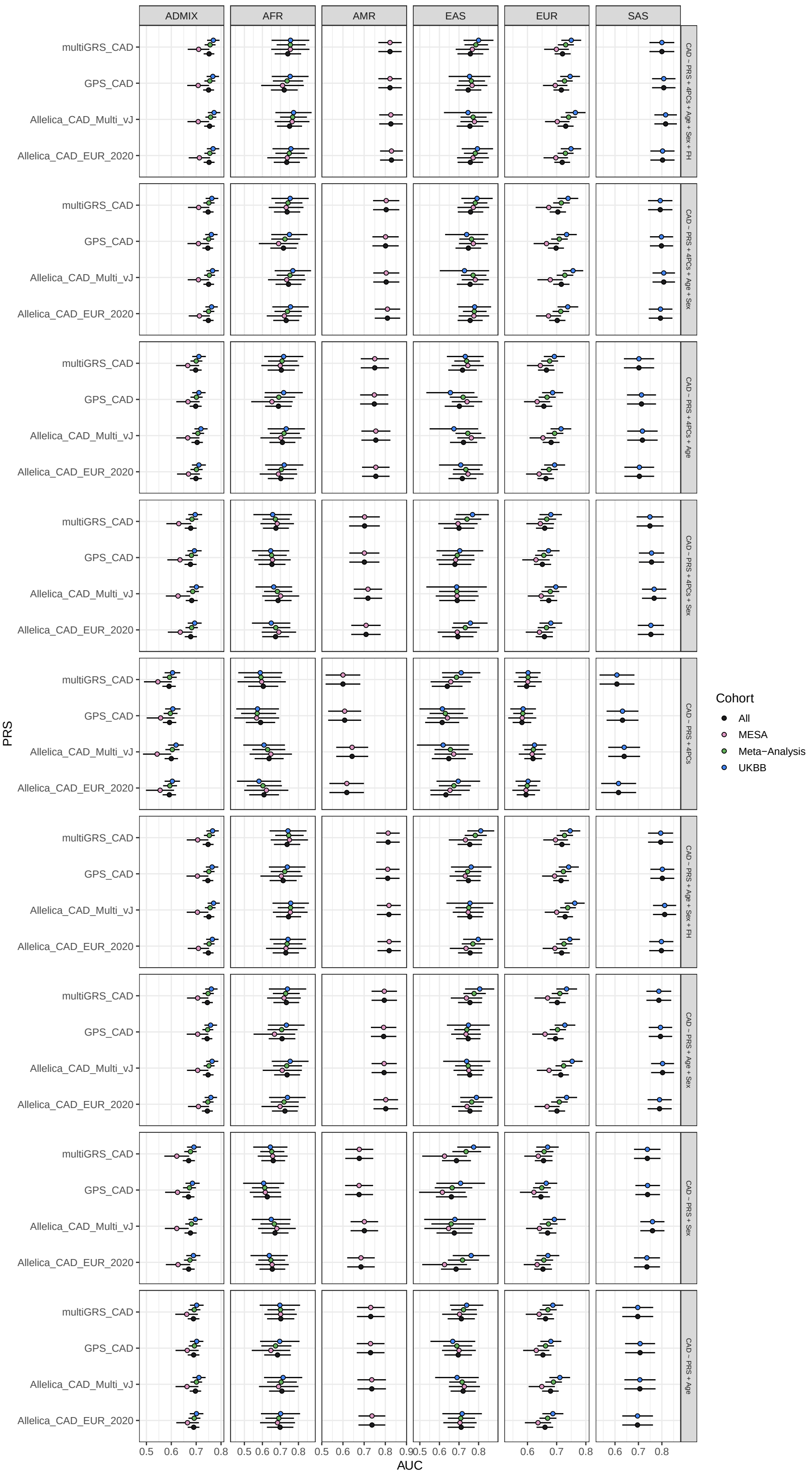

Supplementary Figure 4: Benchmarking of the Allelica_CAD_Multi_vJ PRS scores with three published scores. For each genetic ancestry group, we show the point estimate of the effect size (ORxSD) of the PRS term and 95%CI estimated with the (glm) function in R, for a range of logistic regression models (rows) adjusted for different covariates. The covariates included in the model are reported to the right of each panel row. Numbers of individuals in each genetic ancestry group are detailed in Supplementary Table 15.

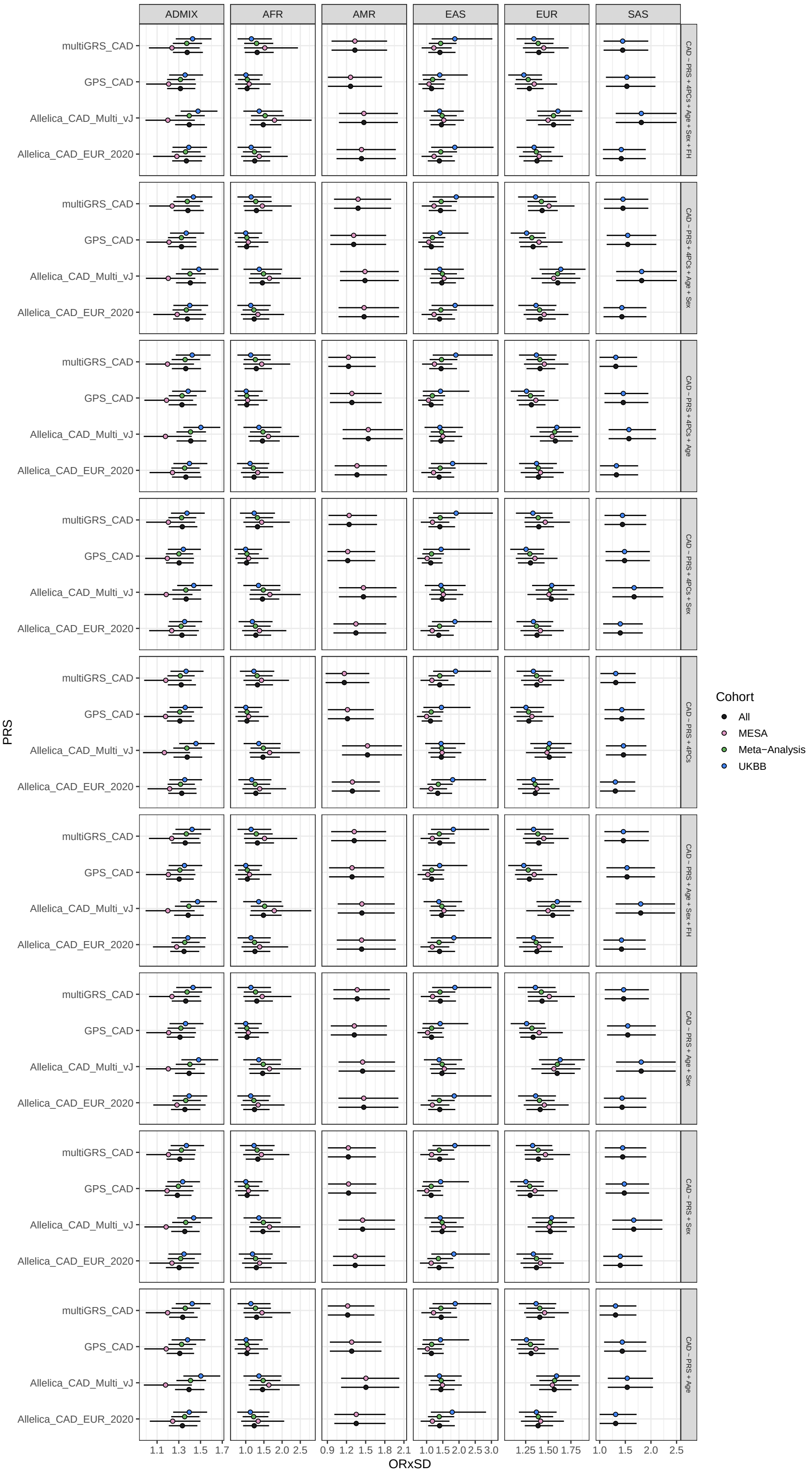

Supplementary Figure 5: Ancestry information for the harmonised dataset. (A) The first and second principal components, computed with 1000 Genomes loadings. The colors represent the ancestries assigned for purposes of deriving ancestry-specific polygenic risk scores. (B) Individual ancestry components as calculated with the iAdmix algorithm. Each row represents a single individual with the fraction of the column colored by the fraction of which they were inferred to be from each ancestry group.

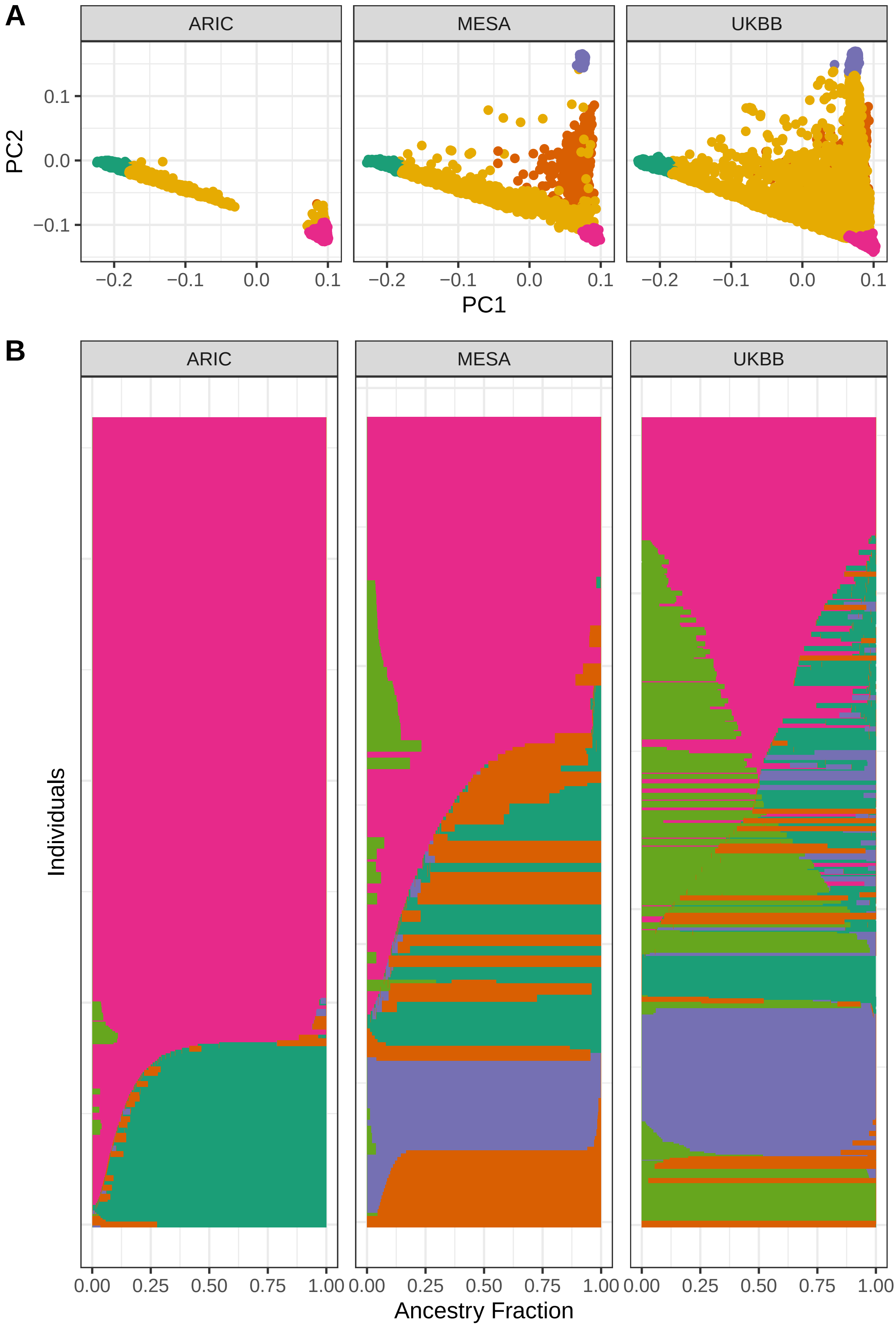

Supplementary Figure 6: Prinicipal Components of Testing Dataset. The top four genetic principal components of individuals in the testing dataset, with points colored by the genetic ancestry inferred by the iAdmix tool.

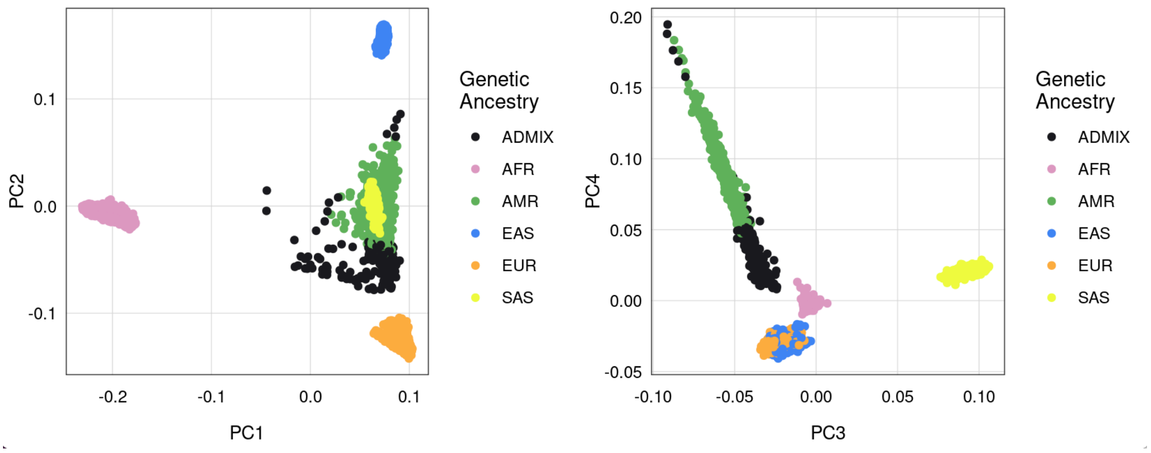

Supplementary Tables

Supplementary Table 1: Labels for the GWASs used in this study. Seven main GWAS were used in this study but were variously filtered based on Minor Allele Frequency (MAF) in relevant the Thousand Genomes Project superpopulation and/or for the top 2 million variants with the lowest P-values

GWAS Name	Source/Ancestry	No. Inds	Variants Retained
EUR-1	CARDIoGRAMplusC4D	184,305	MAF>0.01
EUR-1a	CARDIoGRAMplusC4D	184,305	MAF>0.01; top 2M
EUR-1b	CARDIoGRAMplusC4D	184,305	MAF>0.00001
EUR-2	EUR-1, finemapped	184,305	MAF>0.01
EUR-2a	EUR-1, finemapped	184,305	MAF>0.01; top 2M
EUR-2b	EUR-1, finemapped	184,305	MAF>0.00001
EUR-3	UK Biobank European	459,266	MAF>0.01†
EUR-3a	UK Biobank European	459,266	MAF>0.01†; top 2M
EUR-3b	UK Biobank European	459,266	MAF>0.00001†
EUR-3c	UK Biobank European	459,266	MAF>0.01†
AFR-1	UK Biobank African	3,321	MAF>0.01
EAS-1	Biobank Japan	161,206	MAF>0.01
EAS-2	OACIS (Japan)	52,442	MAF>0.01
SAS-1	UK Biobank South Asian	4,966	MAF>0.01

†:*The MAF in the summary statistics rather than the TGP European superpopulation was used to filter these variants.*

Supplementary Table 2: Datasets used in this study. Note that the case and controls counts for the Genomics and Clinical Datasets may not align with the case and controls counts listed elsewhere in this text because of quality control steps applied to the datasets

Dataset/Cohort	Ancestry	Cases	Controls	Reference
		Genome Wide Association Studies		
CARDIoGRAMplusC4D	European,South Asian	60,801	123,504	Nikpay et al 2015 [1]
UK Biobank European	European	26,135	433,131	Internally Computed
Japanese 161k	East Asian	14,992	146,214	Sakaue et al 2021 [2]
Japanese 52k	East Asian	15,302	36,140	Matsunaga et al 2020 [3]
UK Biobank African	African	91	3,230	Internally Computed
UK Biobank South Asian	South Asian	932	4,043	Internally Computed
**Total**		**118,253**	**746,262**	
		Genomics and Clinical Datasets		
MultiEthnic Study of Atherosclerosis (MESA)	African American, Chinese Hispanic, European	391	5,357	Bild et al 2002 [4]
UK Biobank (UKBB)	African, Admixed South Asian, East Asian	864	11,887	Bycroft et al 2018 [5]
Atherosclerosis Risk in Communities Study (ARIC)	European, African	1,676	9,212	The ARIC Investigators 1989 [6]
**Total**		**2,931**	**26,456**	

Supplementary Table 3: The mean weight derived from the glmnet model for each polygenic risk score and for each ancestry group. An ancestry specific meta polygenic risk score was produced by summing each score (indicated by the first two columns) weighted by the ancestry specific weight (the values in one of the columns in the range 3-7).

PRS-CSx Phi	GWAS Used	AFR	EUR	EAS	SAS	AMR
1E-03	EUR-2	0.0552	0.161	0	0.0677	0.0088
1E-03	EUR-2	0	0	0	0.0237	0
1E-03	EUR-2a	0	0	0.239	0	0
1E-03	EUR-2a	-7.36E-05	0	0.00342	0.000149	0
1E+00	EUR-1_EUR-2_EUR-3_AFR-1	2.14E-05	0	0	0.000178	0
1E-02	EUR-1_EUR-2_EUR-3_AFR-1	0	0	0	0	0
1E-04	EUR-1_EUR-2_EUR-3_AFR-1	0	0.000257	-0.0015	0	0
1E-06	EUR-1_EUR-2_EUR-3_AFR-1	0.0126	0.00238	0	0	0
1E+00	EUR-2a_AFR-1	0.00479	0	0.000573	0	0.0028
1E-02	EUR-2a_AFR-1	0	0	0	0	0.00122
1E-04	EUR-2a_AFR-1	0	0	5.33E-05	0	0
1E-06	EUR-2a_AFR-1	-0.0361	-0.0111	0	0	0
1E+00	EUR-2_AFR-1	0	0	0	0.0126	0
1E-02	EUR-2_AFR-1	0	0	0	0	0
1E-04	EUR-2_AFR-1	-0.00166	0.00947	0	0	0
1E-06	EUR-2_AFR-1	0	-0.000137	0	0	0
1E+00	EUR-3a_AFR-1	0.000257	-0.000621	0	0.00457	0
1E-02	EUR-3a_AFR-1	0	0	0	0.0131	0
1E-04	EUR-3a_AFR-1	-0.00109	0	0	0	0
1E-06	EUR-3a_AFR-1	0	-0.00237	0	0	0
1E+00	EUR-3_AFR-1	0	0	0	0	0
1E-02	EUR-3_AFR-1	0	0	0	0	0.00712
1E-04	EUR-3_AFR-1	0	0.000229	0	0	0
1E-06	EUR-3_AFR-1	-0.00388	-8.52E-05	0.0352	0	5.4E-05
1E+00	EUR-1a_AFR-1a	0.000495	0	0	0	0
1E-02	EUR-1a_AFR-1a	0	0	0	0	0
1E-04	EUR-1a_AFR-1a	0	0	0.00256	0.00181	0
1E-06	EUR-1a_AFR-1a	-0.00299	-0.000675	-0.0344	0	0
1E+00	EUR-1_AFR-1	0.000453	0.000474	0	0.0246	0
1E-02	EUR-1_AFR-1	0	0.0103	0	0	0
1E-04	EUR-1_AFR-1	0	0	0	0	0
1E-06	EUR-1_AFR-1	0.0164	0	0	0.185	0
1E+00	EUR-1_EUR-2_EUR-3_AFR-1_EAS-1_SAS-1	0	0	0	0	0
1E-02	EUR-1_EUR-2_EUR-3_AFR-1_EAS-1_SAS-1	0	0	0	0	0
1E-04	EUR-1_EUR-2_EUR-3_AFR-1_EAS-1_SAS-1	0	0	0.00294	0	0
1E-06	EUR-1_EUR-2_EUR-3_AFR-1_EAS-1_SAS-1	0.0449	0.00161	0	0	0
1E+00	EUR-2_AFR-1_EAS-1_SAS-1	0	4.69E-05	0	0	0
1E-02	EUR-2_AFR-1_EAS-1_SAS-1	0	0.012	0	0	0
1E-04	EUR-2_AFR-1_EAS-1_SAS-1	-0.000319	0.00295	0.00613	0	0
1E-06	EUR-2_AFR-1_EAS-1_SAS-1	0	0.00243	-0.0385	-0.00209	0
1E+00	EUR-1_AFR-1_EAS-1_SAS-1	0	0.00242	0	0	0
1E-02	EUR-1_AFR-1_EAS-1_SAS-1	0	0	0	0	0
1E-04	EUR-1_AFR-1_EAS-1_SAS-1	0	0.000111	0.000502	0	0
1E-06	EUR-1_AFR-1_EAS-1_SAS-1	-6.94E-05	0	0	0	0.000347
1E+00	EUR-3_AFR-1_EAS-1_SAS-1	0	0	0	0	0
1E-02	EUR-3_AFR-1_EAS-1_SAS-1	0	0	0	0	0
1E-04	EUR-3_AFR-1_EAS-1_SAS-1	-0.00216	0	0.0333	0	0
1E-06	EUR-3_AFR-1_EAS-1_SAS-1	0	0	0	0	0
1E+00	EUR-1_EUR-2_EUR-3	0	0.141	0.0119	0.169	0

PRS-CSx Phi	GWAS Used	AFR	EUR	EAS	SAS	AMR
1E-02	EUR-1_EUR-2_EUR-3	6.43E-05	0	0	0.00154	0
1E-04	EUR-1_EUR-2_EUR-3	0	0.00235	0	0	0
1E-06	EUR-1_EUR-2_EUR-3	0	0	-0.00132	0	0
1E+00	EUR-3_EAS-2	-0.000746	-0.00101	0.155	0.000782	0
1E-02	EUR-3_EAS-2	0	-0.000269	0	-4.22E-06	0
1E-04	EUR-3_EAS-2	0.000207	-0.000533	0.00143	-0.0197	0
1E-06	EUR-3_EAS-2	-0.000751	0	0.0436	-0.00184	-0.00097
1E+00	EUR-3_EAS-1	-2.35E-06	-0.000107	0.00214	0.000125	0
1E-02	EUR-3_EAS-1	0	-3.19E-07	0	-3.61E-06	0
1E-04	EUR-3_EAS-1	3.82E-05	-3.62E-05	8.85E-06	-2.97E-05	0
1E-06	EUR-3_EAS-1	-2.54E-07	0	0.000317	-6.61E-08	-9.06E-19
1E+00	EUR-3_SAS-1	0	0	0	0	0
1E-02	EUR-3_SAS-1	0	0	0	0	0
1E-04	EUR-3_SAS-1	0.000604	0	0	0	0
1E-06	EUR-3_SAS-1	0	-0.00272	2.75E-06	-0.00252	0
1E-03	EUR-1a_AFR-1	-0.00095	0	0	-0.00104	0.0565
1E-03	EUR-1a_AFR-1	0	-8.11E-05	0.000862	0	0
1E-03	EUR-1a_AFR-1	0	-0.000221	-0.0056	0	0
1E-03	EUR-1a_AFR-1	0	-0.00738	0	0	0
1E-03	EUR-1a_AFR-1	-0.00255	-1.82E-05	0	0	0
1E-03	EUR-1a_AFR-1	-0.00116	0	-0.00157	-5.24E-05	0
1E-03	EUR-1a_AFR-1	-0.00114	0.00669	0	0	-0.106
1E-03	EUR-1a_AFR-1	-0.000355	0	0	0	0
1E+00	EUR-1_EUR-2_EUR-3_EAS-2	-0.000677	0	0	0	0
1E-02	EUR-1_EUR-2_EUR-3_EAS-2	0	0	0	0	0
1E-04	EUR-1_EUR-2_EUR-3_EAS-2	0.0886	0	0	0	0.000347
1E-06	EUR-1_EUR-2_EUR-3_EAS-2	0	0.00255	0	0.0011	0
1E+00	EUR-2_EUR-3_EAS-2	-0.000423	0	0	0.000368	0
1E-02	EUR-2_EUR-3_EAS-2	0	0	0	0	0
1E-04	EUR-2_EUR-3_EAS-2	0	0	0	0	0
1E-06	EUR-2_EUR-3_EAS-2	0	-0.00138	0	0.00734	0
1E+00	EUR-3_EAS-1	0	0	0.0964	-0.00161	0
1E-02	EUR-3_EAS-1	0	0.000507	0.0289	-0.000851	0.00529
1E-04	EUR-3_EAS-1	0	0.00522	0.0307	0	0
1E-06	EUR-3_EAS-1	0	-0.000435	0	0.00947	0
1E+00	EUR-2_EAS-2	0.000577	0.0896	0	0	0
1E-02	EUR-2_EAS-2	0.061	9.08E-05	0	0	0
1E-04	EUR-2_EAS-2	0.00402	0.000469	0	0	0
1E-06	EUR-2_EAS-2	0	0	0	0.000658	0
1E+00	SAS-1_EUR-1_EUR-2_EUR-3	0	0	0	0	0
1E-02	SAS-1_EUR-1_EUR-2_EUR-3	0	0	0	0	0
1E-04	SAS-1_EUR-1_EUR-2_EUR-3	0	0	0	0	0.00872
1E-06	SAS-1_EUR-1_EUR-2_EUR-3	0.024	0	-8.51E-05	0	0.000116
1E+00	SAS-1_EUR-3_EUR-2	0	0	0	0	0
1E-02	SAS-1_EUR-3_EUR-2	0	0	-0.000471	0	0
1E-04	SAS-1_EUR-3_EUR-2	0	0	-0.00784	0	0
1E-06	SAS-1_EUR-3_EUR-2	1.7E-06	0	-0.00585	0.00344	0
1E+00	SAS-1_EUR-2	0	0	-0.000279	0	0
1E-02	SAS-1_EUR-2	0	0	0	0	0

PRS-CSx Phi	GWAS Used	AFR	EUR	EAS	SAS	AMR
1E-04	SAS-1_EUR-2	0	0	0	0	0
1E-06	SAS-1_EUR-2	0.00323	-0.00187	-0.000389	-0.00169	0.00162
1E+00	SAS-1_EUR-3_EUR-1b	0	0.000565	-0.0596	0	0
1E-02	SAS-1_EUR-3_EUR-1b	7.42E-05	0.00124	0	0.000936	0
1E-04	SAS-1_EUR-3_EUR-1b	0.00989	0.0231	0	0	0
1E-06	SAS-1_EUR-3_EUR-1b	0.083	0.0135	0	0	0
1E+00	AFR-1_EUR-1_EUR-2_EUR-3	0.000432	0	0	0	-0.00455
1E-02	AFR-1_EUR-1_EUR-2_EUR-3	0	0	0	0	0
1E-04	AFR-1_EUR-1_EUR-2_EUR-3	0	0	0.109	-0.00161	0
1E-06	AFR-1_EUR-1_EUR-2_EUR-3	0	0	0.00019	0.00141	0
1E+00	AFR-1_EUR-3_EUR-2a	0	-0.00013	0	0	-0.00647
1E-02	AFR-1_EUR-3_EUR-2a	0	0	0	0	0
1E-04	AFR-1_EUR-3_EUR-2a	0	0	0.00132	0	0
1E-06	AFR-1_EUR-3_EUR-2a	-0.0131	1.67E-05	-0.00566	0.00221	0
1E+00	AFR-1_EUR-3_EUR-2	0	0	-7.01E-05	0.0127	0
1E-02	AFR-1_EUR-3_EUR-2	0	2.01E-05	0	0	0
1E-04	AFR-1_EUR-3_EUR-2	0	0	0	0	0
1E-06	AFR-1_EUR-3_EUR-2	0	-0.00397	-0.00629	0	0
1E+00	AFR-1_EUR-3a	-0.000446	0	0	0	0
1E-02	AFR-1_EUR-3a	0	0	0	0	0
1E-04	AFR-1_EUR-3a	0	0.00452	0.00229	0	0
1E-06	AFR-1_EUR-3a	0	-0.00111	-0.0636	0	0
1E+00	AFR-1_EUR-3	0	0	-0.00748	0.0328	-0.00393
1E-02	AFR-1_EUR-3	0	0	-0.0037	0	0
1E-04	AFR-1_EUR-3	0.000297	0	0	0	0
1E-06	AFR-1_EUR-3	0.00096	-0.0024	0.00639	0	0
1E+00	AFR-1_EUR-3_EUR-1a	0	0	0	0	-0.00442
1E-02	AFR-1_EUR-3_EUR-1a	0	0	0	0	0
1E-04	AFR-1_EUR-3_EUR-1a	-0.0148	6.33E-05	0	-0.00115	0
1E-06	AFR-1_EUR-3_EUR-1a	0.02	0.00138	0.00635	0	0
1E+00	AFR-1_EUR-3_EUR-1	0.0452	0.000271	-0.00367	0	0
1E-02	AFR-1_EUR-3_EUR-1	0	5.12E-05	0	0	0
1E-04	AFR-1_EUR-3_EUR-1	0	0	0.00163	0	0
1E-06	AFR-1_EUR-3_EUR-1	0	0	0.0157	0	0
1E+00	EUR-2b_AFR-1	-0.00573	0	0	0	0
1E-02	EUR-2b_AFR-1	0	0	0	0	0
1E-04	EUR-2b_AFR-1	-0.00233	0	0	0	0
1E-06	EUR-2b_AFR-1	0	-0.000445	0	0.00899	0
1E+00	EUR-3b_AFR-1	-5.08E-05	0	0	-0.000567	0.00126
1E-02	EUR-3b_AFR-1	0	0	0.00533	0	0
1E-04	EUR-3b_AFR-1	0	0	0	0	0
1E-06	EUR-3b_AFR-1	0	-0.00193	-0.00547	-0.0228	0
1E+00	EUR-1b_AFR-1	0	2.35E-05	0	0	0
1E-02	EUR-1b_AFR-1	0	0	0	0	0
1E-04	EUR-1b_AFR-1	0	0	0	0	0
1E-06	EUR-1b_AFR-1	0.0062	-0.000332	-0.000371	0	0
1E+00	EUR-3c_AFR-1	0	0	0	0.061	0.00098
1E-02	EUR-3c_AFR-1	0	0	0.00078	0	0
1E-04	EUR-3c_AFR-1	0	0	0	0	0
1E-06	EUR-3c_AFR-1	-0.00579	0	-0.0195	0	0

Supplementary Table 4: The standard error of the weight derived from the glmnet model for each polygenic risk score and for each ancestry group. An ancestry specific meta polygenic risk score was produced by summing each score (indicated by the first two columns) weighted by the ancestry specific weight (the values in one of the columns in the range 3-7).

PRS-CSx Phi	GWAS Used	AFR	EUR	EAS	SAS	AMR
1E-03	EUR-2	0.00284	0.00236	0	0.0052	0.00299
1E-03	EUR-2	0	0	0	0.00358	0
1E-03	EUR-2a	0	0	0.00679	0	0
1E-03	EUR-2a	7.36E-05	0	0.0017	0.000149	0
1E+00	EUR-1_EUR-2_EUR-3_AFR-1	2.14E-05	0	0	0.000178	0
1E-02	EUR-1_EUR-2_EUR-3_AFR-1	0	0	0	0	0
1E-04	EUR-1_EUR-2_EUR-3_AFR-1	0	0.000232	0.0015	0	0
1E-06	EUR-1_EUR-2_EUR-3_AFR-1	0.00284	0.00106	0	0	0
1E+00	EUR-2a_AFR-1	0.00153	0	0.000556	0	0.00133
1E-02	EUR-2a_AFR-1	0	0	0	0	0.000861
1E-04	EUR-2a_AFR-1	0	0	5.33E-05	0	0
1E-06	EUR-2a_AFR-1	0.00502	0.00213	0	0	0
1E+00	EUR-2_AFR-1	0	0	0	0.00322	0
1E-02	EUR-2_AFR-1	0	0	0	0	0
1E-04	EUR-2_AFR-1	0.00117	0.00159	0	0	0
1E-06	EUR-2_AFR-1	0	0.000102	0	0	0
1E+00	EUR-3a_AFR-1	0.000257	0.000398	0	0.0019	0
1E-02	EUR-3a_AFR-1	0	0	0	0.00275	0
1E-04	EUR-3a_AFR-1	0.000571	0	0	0	0
1E-06	EUR-3a_AFR-1	0	0.000759	0	0	0
1E+00	EUR-3_AFR-1	0	0	0	0	0
1E-02	EUR-3_AFR-1	0	0	0	0	0.00303
1E-04	EUR-3_AFR-1	0	0.000229	0	0	0
1E-06	EUR-3_AFR-1	0.00122	8.52E-05	0.00652	0	5.4E-05
1E+00	EUR-1a_AFR-1a	0.000464	0	0	0	0
1E-02	EUR-1a_AFR-1a	0	0	0	0	0
1E-04	EUR-1a_AFR-1a	0	0	0.00129	9E-04	0
1E-06	EUR-1a_AFR-1a	0.00146	0.000347	0.00734	0	0
1E+00	EUR-1_AFR-1	0.000387	0.000388	0	0.00375	0
1E-02	EUR-1_AFR-1	0	0.00145	0	0	0
1E-04	EUR-1_AFR-1	0	0	0	0	0
1E-06	EUR-1_AFR-1	0.0036	0	0	0.00511	0
1E+00	EUR-1_EUR-2_EUR-3_AFR-1_EAS-1_SAS-1	0	0	0	0	0
1E-02	EUR-1_EUR-2_EUR-3_AFR-1_EAS-1_SAS-1	0	0	0	0	0
1E-04	EUR-1_EUR-2_EUR-3_AFR-1_EAS-1_SAS-1	0	0	0.00172	0	0
1E-06	EUR-1_EUR-2_EUR-3_AFR-1_EAS-1_SAS-1	0.00318	0.000434	0	0	0
1E+00	EUR-2_AFR-1_EAS-1_SAS-1	0	4.69E-05	0	0	0
1E-02	EUR-2_AFR-1_EAS-1_SAS-1	0	0.00132	0	0	0
1E-04	EUR-2_AFR-1_EAS-1_SAS-1	0.000319	0.00074	0.00402	0	0
1E-06	EUR-2_AFR-1_EAS-1_SAS-1	0	0.000754	0.00946	0.00153	0
1E+00	EUR-1_AFR-1_EAS-1_SAS-1	0	0.000687	0	0	0
1E-02	EUR-1_AFR-1_EAS-1_SAS-1	0	0	0	0	0
1E-04	EUR-1_AFR-1_EAS-1_SAS-1	0	0.000111	0.000502	0	0
1E-06	EUR-1_AFR-1_EAS-1_SAS-1	6.94E-05	0	0	0	0.000203
1E+00	EUR-3_AFR-1_EAS-1_SAS-1	0	0	0	0	0
1E-02	EUR-3_AFR-1_EAS-1_SAS-1	0	0	0	0	0
1E-04	EUR-3_AFR-1_EAS-1_SAS-1	0.000974	0	0.00787	0	0
1E-06	EUR-3_AFR-1_EAS-1_SAS-1	0	0	0	0	0
1E+00	EUR-1_EUR-2_EUR-3	0	0.00185	0.00296	0.00476	0

PRS-CSx Phi	GWAS Used	AFR	EUR	EAS	SAS	AMR
1E-02	EUR-1_EUR-2_EUR-3	6.43E-05	0	0	0.000942	0
1E-04	EUR-1_EUR-2_EUR-3	0	0.000619	0	0	0
1E-06	EUR-1_EUR-2_EUR-3	0	0	0.000626	0	0
1E+00	EUR-3_EAS-2	0.000448	0.000362	0.00428	0.000706	0
1E-02	EUR-3_EAS-2	0	0.000269	0	4.22E-06	0
1E-04	EUR-3_EAS-2	0.000194	0.000318	0.000756	0.00292	0
1E-06	EUR-3_EAS-2	0.00028	0	0.00445	0.000807	0.00078
1E+00	EUR-3_EAS-1	2.35E-06	7.89E-05	0.000478	0.000124	0
1E-02	EUR-3_EAS-1	0	3.19E-07	0	3.61E-06	0
1E-04	EUR-3_EAS-1	3.82E-05	2.17E-05	7.09E-06	2.46E-05	0
1E-06	EUR-3_EAS-1	2.31E-07	0	0.000103	4.65E-08	9.06E-19
1E+00	EUR-3_SAS-1	0	0	0	0	0
1E-02	EUR-3_SAS-1	0	0	0	0	0
1E-04	EUR-3_SAS-1	0.000393	0	0	0	0
1E-06	EUR-3_SAS-1	0	0.000932	2.75E-06	0.00145	0
1E-03	EUR-1a_AFR-1	0.000466	0	0	0.00052	0.00831
1E-03	EUR-1a_AFR-1	0	8.11E-05	0.000471	0	0
1E-03	EUR-1a_AFR-1	0	0.000186	0.00162	0	0
1E-03	EUR-1a_AFR-1	0	0.00199	0	0	0
1E-03	EUR-1a_AFR-1	0.00074	1.82E-05	0	0	0
1E-03	EUR-1a_AFR-1	0.000314	0	0.00091	5.24E-05	0
1E-03	EUR-1a_AFR-1	0.000748	0.00117	0	0	0.0116
1E-03	EUR-1a_AFR-1	0.000355	0	0	0	0
1E+00	EUR-1_EUR-2_EUR-3_EAS-2	0.000677	0	0	0	0
1E-02	EUR-1_EUR-2_EUR-3_EAS-2	0	0	0	0	0
1E-04	EUR-1_EUR-2_EUR-3_EAS-2	0.00462	0	0	0	0.00025
1E-06	EUR-1_EUR-2_EUR-3_EAS-2	0	0.000943	0	0.000556	0
1E+00	EUR-2_EUR-3_EAS-2	0.000318	0	0	0.000368	0
1E-02	EUR-2_EUR-3_EAS-2	0	0	0	0	0
1E-04	EUR-2_EUR-3_EAS-2	0	0	0	0	0
1E-06	EUR-2_EUR-3_EAS-2	0	0.000729	0	0.00173	0
1E+00	EUR-3_EAS-1	0	0	0.00644	0.00105	0
1E-02	EUR-3_EAS-1	0	0.000228	0.00545	0.000592	0.00193
1E-04	EUR-3_EAS-1	0	0.00115	0.00736	0	0
1E-06	EUR-3_EAS-1	0	0.000307	0	0.00239	0
1E+00	EUR-2_EAS-2	0.000557	0.00141	0	0	0
1E-02	EUR-2_EAS-2	0.00405	7.86E-05	0	0	0
1E-04	EUR-2_EAS-2	0.00124	0.000316	0	0	0
1E-06	EUR-2_EAS-2	0	0	0	0.000657	0
1E+00	SAS-1_EUR-1_EUR-2_EUR-3	0	0	0	0	0
1E-02	SAS-1_EUR-1_EUR-2_EUR-3	0	0	0	0	0
1E-04	SAS-1_EUR-1_EUR-2_EUR-3	0	0	0	0	0.00283
1E-06	SAS-1_EUR-1_EUR-2_EUR-3	0.00277	0	8.51E-05	0	0.000116
1E+00	SAS-1_EUR-3_EUR-2	0	0	0	0	0
1E-02	SAS-1_EUR-3_EUR-2	0	0	0.000471	0	0
1E-04	SAS-1_EUR-3_EUR-2	0	0	0.00353	0	0
1E-06	SAS-1_EUR-3_EUR-2	1.7E-06	0	0.00216	0.00126	0
1E+00	SAS-1_EUR-2	0	0	0.000279	0	0
1E-02	SAS-1_EUR-2	0	0	0	0	0

PRS-CSx Phi	GWAS Used	AFR	EUR	EAS	SAS	AMR
1E-04	SAS-1_EUR-2	0	0	0	0	0
1E-06	SAS-1_EUR-2	0.000943	0.000823	0.000385	0.00114	0.00114
1E+00	SAS-1_EUR-3_EUR-1b	0	0.000349	0.00629	0	0
1E-02	SAS-1_EUR-3_EUR-1b	7.42E-05	0.000543	0	0.000533	0
1E-04	SAS-1_EUR-3_EUR-1b	0.00161	0.00156	0	0	0
1E-06	SAS-1_EUR-3_EUR-1b	0.0038	0.00212	0	0	0
1E+00	AFR-1_EUR-1_EUR-2_EUR-3	0.000319	0	0	0	0.00225
1E-02	AFR-1_EUR-1_EUR-2_EUR-3	0	0	0	0	0
1E-04	AFR-1_EUR-1_EUR-2_EUR-3	0	0	0.00899	0.00152	0
1E-06	AFR-1_EUR-1_EUR-2_EUR-3	0	0	0.00019	0.000934	0
1E+00	AFR-1_EUR-3_EUR-2a	0	0.00013	0	0	0.00276
1E-02	AFR-1_EUR-3_EUR-2a	0	0	0	0	0
1E-04	AFR-1_EUR-3_EUR-2a	0	0	0.000805	0	0
1E-06	AFR-1_EUR-3_EUR-2a	0.00279	1.67E-05	0.00198	0.000916	0
1E+00	AFR-1_EUR-3_EUR-2	0	0	7.01E-05	0.00272	0
1E-02	AFR-1_EUR-3_EUR-2	0	2.01E-05	0	0	0
1E-04	AFR-1_EUR-3_EUR-2	0	0	0	0	0
1E-06	AFR-1_EUR-3_EUR-2	0	0.00104	0.00267	0	0
1E+00	AFR-1_EUR-3a	0.000446	0	0	0	0
1E-02	AFR-1_EUR-3a	0	0	0	0	0
1E-04	AFR-1_EUR-3a	0	0.00115	0.00136	0	0
1E-06	AFR-1_EUR-3a	0	0.000448	0.0105	0	0
1E+00	AFR-1_EUR-3	0	0	0.00282	0.0036	0.00193
1E-02	AFR-1_EUR-3	0	0	0.00221	0	0
1E-04	AFR-1_EUR-3	0.000297	0	0	0	0
1E-06	AFR-1_EUR-3	0.000581	0.000808	0.00272	0	0
1E+00	AFR-1_EUR-3_EUR-1a	0	0	0	0	0.00189
1E-02	AFR-1_EUR-3_EUR-1a	0	0	0	0	0
1E-04	AFR-1_EUR-3_EUR-1a	0.00321	6.33E-05	0	0.00115	0
1E-06	AFR-1_EUR-3_EUR-1a	0.00345	0.000499	0.00288	0	0
1E+00	AFR-1_EUR-3_EUR-1	0.0046	0.000155	0.00168	0	0
1E-02	AFR-1_EUR-3_EUR-1	0	5.12E-05	0	0	0
1E-04	AFR-1_EUR-3_EUR-1	0	0	0.00159	0	0
1E-06	AFR-1_EUR-3_EUR-1	0	0	0.0041	0	0
1E+00	EUR-2b_AFR-1	0.00201	0	0	0	0
1E-02	EUR-2b_AFR-1	0	0	0	0	0
1E-04	EUR-2b_AFR-1	0.000952	0	0	0	0
1E-06	EUR-2b_AFR-1	0	0.000335	0	0.00284	0
1E+00	EUR-3b_AFR-1	4.58E-05	0	0	0.000567	0.00126
1E-02	EUR-3b_AFR-1	0	0	0.00203	0	0
1E-04	EUR-3b_AFR-1	0	0	0	0	0
1E-06	EUR-3b_AFR-1	0	0.000686	0.00238	0.00369	0
1E+00	EUR-1b_AFR-1	0	2.35E-05	0	0	0
1E-02	EUR-1b_AFR-1	0	0	0	0	0
1E-04	EUR-1b_AFR-1	0	0	0	0	0
1E-06	EUR-1b_AFR-1	0.002	0.000268	0.000371	0	0
1E+00	EUR-3c_AFR-1	0	0	0	0.0055	0.00098
1E-02	EUR-3c_AFR-1	0	0	0.00078	0	0
1E-04	EUR-3c_AFR-1	0	0	0	0	0
1E-06	EUR-3c_AFR-1	0.00147	0	0.00521	0	0

Supplementary Table 5: Performance statistics for the multi-ancestry PRSs and the three benchmarking scores. For each of the best multi-ancestry scores and the three external PRS for comparison PRS we show: the Brier scores, a Brier score of 0 equates to perfectly calibrated model and 1 is perfectly uncalibrated; the area under the receiver operator curve (AUC) measured through predictions generated by a logistic regression model that also included the covariates of the first four principal components, age, sex, family history of disease and cohort; the Nagelkerke's R^2 value measured through predictions generated by a logistic regression model that also included the covariates of the first four principal components, age, sex, family history of disease and cohort; and the 2X Thresholds at which individuals in the high risk tail of the distribution have twice the odds of CAD as those in the remainder of the distribution. Confidence intervals for these thresholds are provided in the following table. The methods employed to determine these thresholds and confidence intervals are provided in the methods section.

Ancestry	Cohort	PRS	ORxSD (95% CI)	Brier Score	AUC (95% CI)	R2	OR 2X Thresh. Percentile
African	MESA	Allelica_CAD_Multi_vJ	1.79 (1.14 - 2.81)	0.04167	0.765 (0.668 - 0.861)	0.149	85
African	UKBB	Allelica_CAD_Multi_vJ	1.37 (0.935 - 2.01)	0.03172	0.773 (0.672 - 0.873)	0.131	85
African	META	Allelica_CAD_Multi_vJ	1.53 (1.15 - 2.05)	0.03618	0.768 (0.698 - 0.847)	0.139	85
American	MESA	Allelica_CAD_Multi_vJ	1.47 (1.08 - 2.01)	0.05918	0.826 (0.771 - 0.882)	0.234	88
East Asian	MESA	Allelica_CAD_Multi_vJ	1.53 (1.09 - 2.15)	0.03899	0.779 (0.707 - 0.851)	0.156	86
East Asian	UKBB	Allelica_CAD_Multi_vJ	1.4 (0.913 - 2.15)	0.02567	0.746 (0.623 - 0.87)	0.111	86
East Asian	META	Allelica_CAD_Multi_vJ	1.48 (1.13 - 1.93)	0.03388	0.772 (0.708 - 0.841)	0.139	86
European	MESA	Allelica_CAD_Multi_vJ	1.5 (1.26 - 1.78)	0.07023	0.702 (0.66 - 0.744)	0.0934	83
European	UKBB	Allelica_CAD_Multi_vJ	1.61 (1.38 - 1.88)	0.07505	0.764 (0.73 - 0.799)	0.159	83
European	META	Allelica_CAD_Multi_vJ	1.56 (1.39 - 1.75)	0.07298	0.741 (0.714 - 0.769)	0.131	83
South Asian	UKBB	Allelica_CAD_Multi_vJ	1.81 (1.31 - 2.5)	0.09262	0.816 (0.768 - 0.864)	0.272	76
African	MESA	GPS_CAD	1.09 (0.71 - 1.69)	0.04219	0.712 (0.593 - 0.83)	0.11	95
African	UKBB	GPS_CAD	1 (0.689 - 1.46)	0.03185	0.754 (0.652 - 0.856)	0.119	NA
African	META	GPS_CAD	1.04 (0.785 - 1.39)	0.03648	0.737 (0.658 - 0.826)	0.115	95
American	MESA	GPS_CAD	1.26 (0.908 - 1.76)	0.06003	0.822 (0.767 - 0.877)	0.22	94
East Asian	MESA	GPS_CAD	1.08 (0.748 - 1.55)	0.04009	0.767 (0.689 - 0.845)	0.13	94
East Asian	UKBB	GPS_CAD	1.41 (0.87 - 2.27)	0.02581	0.754 (0.647 - 0.861)	0.109	NA
East Asian	META	GPS_CAD	1.19 (0.888 - 1.58)	0.0346	0.763 (0.698 - 0.833)	0.122	94
European	MESA	GPS_CAD	1.34 (1.13 - 1.6)	0.07086	0.695 (0.653 - 0.737)	0.0819	93
European	UKBB	GPS_CAD	1.23 (1.06 - 1.43)	0.07712	0.746 (0.712 - 0.78)	0.131	93
European	META	GPS_CAD	1.28 (1.14 - 1.43)	0.07443	0.727 (0.701 - 0.755)	0.11	93
South Asian	UKBB	GPS_CAD	1.53 (1.12 - 2.09)	0.09441	0.808 (0.758 - 0.858)	0.252	77
African	MESA	Allelica_CAD_EUR_2020	1.37 (0.874 - 2.15)	0.04255	0.738 (0.627 - 0.849)	0.121	95
African	UKBB	Allelica_CAD_EUR_2020	1.15 (0.774 - 1.7)	0.03182	0.758 (0.656 - 0.86)	0.121	NA
African	META	Allelica_CAD_EUR_2020	1.24 (0.922 - 1.67)	0.03663	0.749 (0.672 - 0.835)	0.121	95
American	MESA	Allelica_CAD_EUR_2020	1.43 (1.04 - 1.97)	0.05982	0.829 (0.775 - 0.883)	0.23	94
East Asian	MESA	Allelica_CAD_EUR_2020	1.23 (0.843 - 1.8)	0.03989	0.772 (0.69 - 0.853)	0.135	94
East Asian	UKBB	Allelica_CAD_EUR_2020	1.87 (1.14 - 3.07)	0.0257	0.794 (0.714 - 0.874)	0.137	NA
East Asian	META	Allelica_CAD_EUR_2020	1.44 (1.06 - 1.94)	0.03444	0.783 (0.725 - 0.846)	0.136	94
European	MESA	Allelica_CAD_EUR_2020	1.4 (1.18 - 1.66)	0.07065	0.697 (0.655 - 0.739)	0.0859	95
European	UKBB	Allelica_CAD_EUR_2020	1.34 (1.15 - 1.57)	0.07649	0.749 (0.714 - 0.784)	0.138	95
European	META	Allelica_CAD_EUR_2020	1.37 (1.22 - 1.53)	0.07397	0.73 (0.703 - 0.758)	0.115	95
South Asian	UKBB	Allelica_CAD_EUR_2020	1.42 (1.06 - 1.9)	0.09451	0.803 (0.751 - 0.854)	0.247	94
African	MESA	multiGRS_CAD	1.52 (0.953 - 2.44)	0.0426	0.755 (0.649 - 0.861)	0.129	95
African	UKBB	multiGRS_CAD	1.16 (0.776 - 1.72)	0.0318	0.756 (0.651 - 0.86)	0.121	NA
African	META	multiGRS_CAD	1.3 (0.958 - 1.76)	0.03663	0.755 (0.679 - 0.84)	0.124	95
American	MESA	multiGRS_CAD	1.33 (0.965 - 1.84)	0.05985	0.822 (0.767 - 0.877)	0.224	94
East Asian	MESA	multiGRS_CAD	1.22 (0.846 - 1.77)	0.0398	0.768 (0.683 - 0.853)	0.135	94
East Asian	UKBB	multiGRS_CAD	1.87 (1.16 - 3.03)	0.02588	0.8 (0.723 - 0.876)	0.139	NA
East Asian	META	multiGRS_CAD	1.43 (1.07 - 1.92)	0.03445	0.786 (0.728 - 0.848)	0.136	94
European	MESA	multiGRS_CAD	1.45 (1.22 - 1.72)	0.07033	0.699 (0.657 - 0.742)	0.0899	94
European	UKBB	multiGRS_CAD	1.34 (1.15 - 1.56)	0.07659	0.75 (0.716 - 0.784)	0.138	94
European	META	multiGRS_CAD	1.39 (1.24 - 1.56)	0.0739	0.731 (0.704 - 0.759)	0.117	94
South Asian	UKBB	multiGRS_CAD	1.45 (1.08 - 1.95)	0.09373	0.8 (0.747 - 0.853)	0.248	77

Supplementary Table 6: Brier scores calculated upon individuals who were not assigned to any of the single ancestry groups with predictions from models fit to the single ancestry groups. The predictions were weighted by each individual's iAdmix ancestry component and log(ORxSD) of the single ancestry to create a weighted average prediction.

Dataset	Cohort	PRS	Brier Score
Full Dataset	MESA	Allelica_CAD_Multi_vJ	0.06308
Full Dataset	UKBB	Allelica_CAD_Multi_vJ	0.05827
Full Dataset	META	Allelica_CAD_Multi_vJ	0.06085
Full Dataset	MESA	GPS_CAD	0.06265
Full Dataset	UKBB	GPS_CAD	0.05851
Full Dataset	META	GPS_CAD	0.06089
Full Dataset	MESA	Allelica_CAD_EUR_2020	0.06278
Full Dataset	UKBB	Allelica_CAD_EUR_2020	0.05854
Full Dataset	META	Allelica_CAD_EUR_2020	0.06095
Full Dataset	MESA	multiGRS_CAD	0.06287
Full Dataset	UKBB	multiGRS_CAD	0.05853
Full Dataset	META	multiGRS_CAD	0.06107

Supplementary Table 7: Comparison between each of the Allelica_CAD_Multi_vJ PRSs and the Next Best Competing PRS. For each ancestry group the competing PRS (among GPS_CAD, Allelica_CAD_EUR_2020, and multiGRS_CAD) with the greatest ORxSD and its statistics were directly compared to the statistics of each of the Allelica_CAD_Multi_vJ PRSs.

Ancestry	Allelica_CAD_Multi_vJ ORxSD (95% CI)	Allelica_CAD_Multi_vJ Brier Score	Best Competing PRS ORxSD (95% CI)	Best Competing PRS Brier Score	% Improvement in ORxSD	Brier Skill Score
African	1.53 (1.15 - 2.05)	0.0362	1.3 (0.958 - 1.76)	0.03645	18.2	0.0076
American	1.47 (1.08 - 2.01)	0.0592	1.43 (1.04 - 1.97)	0.05982	2.61	0.0104
East Asian	1.48 (1.13 - 1.93)	0.0339	1.44 (1.06 - 1.94)	0.03444	3.02	0.0158
European	1.56 (1.39 - 1.75)	0.0730	1.39 (1.24 - 1.56)	0.07390	12.2	0.0121
South Asian	1.81 (1.31 - 2.50)	0.0926	1.53 (1.12 - 2.09)	0.09373	18.4	0.0120

Supplementary Table 8: Odds Ratio Threshold statistics for the multi-ancestry PRSs and the three benchmarking scores. The 2X Thresholds at which individuals in the high risk tail of the distribution have twice the odds of CAD as those in the remainder of the distribution. Additionally, at these threshold we provide the confidence intervals of both the odds ratio and the percentile.

Ancestry	Cohort	PRS	OR 2X Thresh. Percentile	95% CI of OR at 2X Thresh. Percentile	Low 95% CI of Thresh. Percentile	High 95% CI of Thresh. Percentile
African	MESA	Allelica_CAD_Multi_vJ	85	1.17-3.41	76	90
African	UKBB	Allelica_CAD_Multi_vJ	85	1.39-2.88	80	89
African	META	Allelica_CAD_Multi_vJ	85	1.04-3.83	73	91
American	MESA	Allelica_CAD_Multi_vJ	88	1.52-2.63	69	97
East Asian	MESA	Allelica_CAD_Multi_vJ	86	1.36-2.94	70	95
East Asian	UKBB	Allelica_CAD_Multi_vJ	86	1.21-3.31	65	97
East Asian	META	Allelica_CAD_Multi_vJ	86	1.04-3.84	60	97
European	MESA	Allelica_CAD_Multi_vJ	83	1.85-2.16	80	86
European	UKBB	Allelica_CAD_Multi_vJ	83	1.89-2.12	81	85
European	META	Allelica_CAD_Multi_vJ	83	1.55-2.58	72	93
South Asian	UKBB	Allelica_CAD_Multi_vJ	76	1.73-2.32	72	80
African	MESA	GPS_CAD	95	0.859-4.66	62	NA
African	UKBB	GPS_CAD	NA	NA	NA	NA
African	META	GPS_CAD	95	0.766-5.22	60	NA
American	MESA	GPS_CAD	94	1.22-3.27	60	97
East Asian	MESA	GPS_CAD	94	1.19-3.37	64	97
East Asian	UKBB	GPS_CAD	NA	NA	NA	NA
East Asian	META	GPS_CAD	94	0.825-4.85	60	97
European	MESA	GPS_CAD	93	1.72-2.32	76	NA
European	UKBB	GPS_CAD	93	1.8-2.22	78	NA
European	META	GPS_CAD	93	1.41-2.83	68	96
South Asian	UKBB	GPS_CAD	77	1.72-2.32	72	80
African	MESA	Allelica_CAD_EUR_2020	95	0.859-4.66	62	NA
African	UKBB	Allelica_CAD_EUR_2020	NA	NA	NA	NA
African	META	Allelica_CAD_EUR_2020	95	0.766-5.22	60	NA
American	MESA	Allelica_CAD_EUR_2020	94	1.22-3.27	60	97
East Asian	MESA	Allelica_CAD_EUR_2020	94	1.19-3.37	64	97
East Asian	UKBB	Allelica_CAD_EUR_2020	NA	NA	NA	NA
East Asian	META	Allelica_CAD_EUR_2020	94	0.825-4.85	60	97
European	MESA	Allelica_CAD_EUR_2020	95	1.67-2.4	75	NA
European	UKBB	Allelica_CAD_EUR_2020	95	1.72-2.32	76	NA
European	META	Allelica_CAD_EUR_2020	95	1.34-2.98	66	97
South Asian	UKBB	Allelica_CAD_EUR_2020	94	1.4-2.87	66	NA
African	MESA	multiGRS_CAD	95	0.859-4.66	62	NA
African	UKBB	multiGRS_CAD	NA	NA	NA	NA
African	META	multiGRS_CAD	95	0.766-5.22	60	NA
American	MESA	multiGRS_CAD	94	1.22-3.27	60	97
East Asian	MESA	multiGRS_CAD	94	1.19-3.37	64	97
East Asian	UKBB	multiGRS_CAD	NA	NA	NA	NA
East Asian	META	multiGRS_CAD	94	0.825-4.85	60	97
European	MESA	multiGRS_CAD	94	1.71-2.34	76	NA
European	UKBB	multiGRS_CAD	94	1.74-2.3	77	NA
European	META	multiGRS_CAD	94	1.37-2.93	67	97
South Asian	UKBB	multiGRS_CAD	77	1.72-2.32	72	80

Supplementary Table 9: Reclassification of borderline/intermediate 10 year CHD risk in individuals with high PRS. The one-sided P-value for the Net Reclassification Improvement statistic assesses how statistically different the estimate is from 0 and was obtained by dividing the estimated NRI by its standard error and comparing this to the standard normal distribution. The adjusted P-value (Adj. P) is based on a Bonferonni Correction for multiple tests (n = 3 tests).

**Cohort description**	**UKBB Testing Data**	**MESA Testing Data**	**UKBB + MESA Testing Data**
Total individuals	5520	3594	9114
Borderline/Intermediate (BIR) 10 yr PCE Risk (%)	1069 (19.37)	1420 (39.51)	2452 (26.9)
Cases in BIR (%)	105 (9.82)	139 (9.79)	253 (10.32)
Case rate in BIR	9.01	10.62	10.24
BIR Individuals reclassified with High PRS (%)	169 (15.81)	226 (15.92)	402 (16.39)
Median follow-up (years)	11.68	10.31	11.05
Cases in reclassified (%)	31 (18.34)	30 (13.27)	68 (16.92)
Proportion of Cases in BIR (%)	2.9	2.11	2.77
Proportion of reclassified cases over all cases in BIR	0.3	0.22	0.27
Case rate in reclassified	17.43	14.55	17.37
BIR Individuals with Not-elevated PRS (%)	900 (16.3)	1194 (33.22)	2050 (22.49)
Cases in not-reclassified (%)	74 (8.22)	109 (9.13)	185 (9.02)
Case rate in not-reclassified BIR	7.49	9.89	8.89
Relative risk (case %)	2.23	1.45	1.88
Relative risk (case rate)	2.33	1.47	1.95
Total Categorical NRI (95% CI)	0.1193 (0.0638 - 0.1748)	0.0862 (0.037 - 0.1354)	0.1314 (0.0923 - 0.1706)
P value	3.00E-05	5.90E-04	<1e-10
Adj. P value			<1e-10

Supplementary Table 10: Reclassification of borderline/intermediate 10 year ASCVD risk in individuals with high PRS. The one-sided P-value for the Net Reclassification Improvement statistic assesses how statistically different the estimate is from 0 and was obtained by dividing the estimated NRI by its standard error and comparing this to the standard normal distribution. The adjusted P-value (Adj. P) is based on a Bonferonni Correction for multiple tests (n = 3 tests).

**Cohort Description**	**UKBB Testing Data**	**MESA Testing Data**	**UKBB + MESA Testing Data**
Total individuals	5398	3594	8992
Borderline/Intermediate (BIR) 10 yr PCE Risk (%)	1381 (25.58)	1772 (49.3)	3259 (36.24)
Cases in BIR (%)	157 (11.37)	171 (9.65)	362 (11.11)
Case rate in BIR	10.35	10.28	10.97
BIR Individuals reclassified with High PRS (%)	216 (15.64)	289 (16.31)	526 (16.14)
Median follow-up (years)	11.67	10.29	11.04
Cases in reclassified (%)	35 (16.2)	39 (13.49)	86 (16.35)
Proportion of Cases in BIR (%)	2.53	2.2	2.64
Proportion of reclassified cases over all cases in BIR	0.22	0.23	0.24
Case rate in reclassified	15	14.56	16.5
BIR Individuals with Not-elevated PRS (%)	1165 (21.58)	1483 (41.26)	2733 (30.39)
Cases in not-reclassified (%)	122 (10.47)	132 (8.9)	276 (10.1)
Case rate in not-reclassified BIR	9.5	9.46	9.93
Relative risk (case %)	1.55	1.52	1.62
Relative risk (case rate)	1.58	1.54	1.66
Total Categorical NRI (95% CI)	0.0849 (0.0389 - 0.1309)	0.0641 (0.022 - 0.1063)	0.1070 (0.0735 - 0.1405)
P value	2.90E-04	2.87E-03	<1e-10
Adj. P value			<1e-10

Supplementary Table 11: Results of logistic regression for BIR dataset individuals with and without PRS as a risk enhancing factor for CAD. P-values were estimated using the glm function in R and adjusted P-values (Adj. P) are based on a Bonferonni Correction for multiple tests (n = 15 tests).

	**OR (95% CI)**	**P**	**Adj. P**
constant	0.04 (0.03 - 0.07)	<1e-10	<1e-10
Age	1.35 (1.14 - 1.61)	0.001	0.015
Sex	1.43 (0.99 - 2.05)	0.057	0.855
Smoker	1.28 (0.89 - 1.84)	0.178	1.0
LDL-C	1.11 (0.96 - 1.29)	0.159	1.0
HDL-C	0.75 (0.63 - 0.89)	0.001	0.015
Family History	1.36 (1.03 - 1.78)	0.03	0.45
On blood pressure lowering medication	1.39 (1.03 - 1.86)	0.03	0.45
Triglyceride	0.94 (0.8 - 1.1)	0.447	1.0
Diabetes Mellitus	2.07 (1.45 - 2.97)	<1e-10	<1e-10
PRS >2 fold risk threshold	2.03 (1.49 - 2.76)	<1e-10	<1e-10
PC1	1.11 (0.94 - 1.3)	0.224	1.0
PC2	0.81 (0.68 - 0.96)	0.018	0.27
PC3	1.06 (0.93 - 1.21)	0.368	1.0
PC4	1.05 (0.91 - 1.21)	0.519	1.0

Supplementary Table 12: Results of logistic regression for BIR dataset individuals with and without PRS as a risk enhancing factor for ASCVD. P-values were estimated using the glm function in R and adjusted P-values (Adj. P) are based on a Bonferonni Correction for multiple tests (n = 15 tests).

	**OR (95% CI)**	**P**	**Adj. P**
constant	0.05 (0.04 - 0.07)	<1e-10	<1e-10
Age	1.42 (1.23 - 1.63)	<1e-10	<1e-10
Sex	1.41 (1.07 - 1.84)	0.013	0.195
Smoker	1.47 (1.08 - 1.99)	0.013	0.195
LDL-C	1.1 (0.98 - 1.24)	0.119	1.0
HDL-C	0.74 (0.64 - 0.87)	<1e-10	<1e-10
Family History	1.37 (1.09 - 1.72)	0.007	0.105
On blood pressure lowering medication	1.55 (1.21 - 1.98)	<1e-10	<1e-10
Triglyceride	0.96 (0.84 - 1.1)	0.556	1.0
Diabetes Mellitus	2.02 (1.48 - 2.76)	<1e-10	<1e-10
PRS >2 fold risk threshold	1.72 (1.32 - 2.25)	<1e-10	<1e-10
PC1	1.05 (0.92 - 1.2)	0.447	1.0
PC2	0.88 (0.76 - 1.0)	0.054	0.81
PC3	1.02 (0.91 - 1.14)	0.711	1.0
PC4	1.05 (0.94 - 1.18)	0.406	1.0

Supplementary Table 13: The Net Reclassification Improvement (NRI) results when treating the polygenic risk score as a continuous variable using INTERGRATEpce. The one-sided P-value for the Net Reclassification Improvement statistic assesses how statistically different the estimate is from 0 and was obtained by dividing the estimated NRI by its standard error and comparing this to the standard normal distribution. The adjusted P-value (Adj. P) is based on a Bonferonni Correction for multiple tests (n = 12 tests).

Outcome	Genetic Ancestry	Total Ind.	Controls Up	Controls Down	Cases Up	Cases Down	NRI (95% CI)	P	Adj. P
CAD	African	1852	11	2	0	0	-0.005 (-0.009 - -0.0011)	0.0124	0.149
CAD	American	481	7	3	2	0	0.0653 (-0.0345 - 0.1650)	0.2	1.0
CAD	East Asian	991	9	1	3	0	0.1117 (-0.0158 - 0.2393)	0.086	1.0
CAD	European	5151	29	11	12	2	0.0431 (0.0093 - 0.0769)	0.0124	1.0
CAD	South Asian	639	3	4	2	0	0.0731 (-0.0227 - 0.1688)	0.1348	1.0
CAD	ALL	9114	59	21	19	2	0.0431 (0.0185 - 0.0678)	0.0006	0.007
ASCVD	African	1835	4	3	4	0	0.0398 (0.0009 - 0.0787)	0.0448	0.538
ASCVD	American	481	6	4	4	0	0.1007 (0.0022 - 0.1993)	0.0452	0.542
ASCVD	East Asian	985	11	1	1	0	0.0229 (-0.0418 - 0.0875)	0.4881	1.0
ASCVD	European	5065	53	18	12	7	0.0114 (-0.0207 - 0.0435)	0.4852	1.0
ASCVD	South Asian	626	3	7	2	0	0.0639 (-0.0137 - 0.1415)	0.1065	1.28
ASCVD	ALL	8992	77	33	23	7	0.029 (0.0061 - 0.0518)	0.0129	0.155

Supplementary Table 14: The codes used to define an individual within the UK Biobank as a positive case for CAD. An individual who did not have any of these codes was recorded as a negative control.

Outcome-defining UKBB Field	Time-defining UKBB Field	Event Code
Primary and secondary Diagnoses - ICD10 codes (41270)	41280	I21 to I24 or I25.2
Primary and secondary Diagnoses - ICD9 codes (41271)	41281	410 to 412
Operative procedures - OPCS4 (41272)	41282	K40 to K46, K49, K50.1, K50.2, K50.4 or K75
Non-cancer illness code, self-reported (20002)	20008	1075
Self reported Operation code (20004)	20010	1070, 1095, or 1523
Vascular/heart problems diagnosed by doctor (6150)	3894	1
Underlying primary and secondary cause of death: ICD10	40001, 40002	I21 to I24 or I25.2

Supplementary Table 15: The number of cases and controls within the ancestry groups and datasets used to independently validate and test ancestry specific PRS. - Each of the ancestry groups is defined as as genetic similarity to individuals of the 1000 Genomes Projects of a given continental superpopulation. For example, the American ancestry group indcludes individuals who are genetically similar to those in 1000 Genomes Project who were labeled with the ”AMR” superpopulation. More information on the ancestry assignment is provided in both the methods and discussion sections.

**Ancestry**	**Cohort**	**Validation**		**Testing**	
		**Cases**	**Controls**	**Cases**	**Controls**
Admixed	TOTAL	0	0	469	6480
Admixed	MESA	0	0	120	1629
Admixed	UKBB	0	0	349	4851
African	TOTAL	336	2077	51	1248
African	ARIC	336	2077	0	0
African	MESA	0	0	23	464
African	UKBB	0	0	28	784
American	TOTAL	29	630	50	642
American	MESA	19	244	50	642
American	UKBB	10	385	0	0
East Asian	TOTAL	67	1790	50	1343
East Asian	MESA	0	0	31	674
East Asian	UKBB	67	1790	19	669
European	TOTAL	1340	7135	346	3694
European	ARIC	1340	7135	0	0
European	MESA	0	0	148	1704
European	UKBB	0	0	198	1990
South Asian	TOTAL	124	948	69	471
South Asian	UKBB	124	947	69	471

Supplementary Table 16: Comprehensive information for the multi-ancestry PRSs in the Testing datasets. We show the PRS Name, the GWASs used in the development of the PRS, the number of SNPs in the PRS (No. SNPs), the Odds Ratios per Standard Deviation and 95% confidence interval (ORxSD) of the PRS measured with a logistic regression adjusted by the first four principal components, age, sex, family history of disease and cohort, and the percentile threshold where individuals above are at 2 times risk in each Testing dataset (2X Risk) measured relative to all remaining individuals. The 95%CI of the risk at this threshold is also shown (95%CI of 2X Risk).

PRS	GWAS Employed	No. SNPs	ORxSD (95% CI)	AUC (95% CI)	Brier Score
Allelica_CAD_AFR_vJ	Meta	5802524	1.53 (1.15 - 2.05)	0.768 (0.698 - 0.847)	0.03618
Allelica_CAD_AMR_vJ	EUR-1A, AFR-1	648	1.47 (1.08 - 2.01)	0.826 (0.771 - 0.882)	0.05918
Allelica_CAD_EAS_vJ	EUR-3, EAS-1	246708	1.48 (1.13 - 1.93)	0.772 (0.708 - 0.841)	0.03388
Allelica_CAD_EUR_vJ	Meta	285228	1.56 (1.39 - 1.75)	0.741 (0.714 - 0.769)	0.07298
Allelica_CAD_SAS_vJ	Meta	6601285	1.81 (1.31 - 2.5)	0.816 (0.768 - 0.864)	0.09262

Supplementary Table 17: Description of the coding languages employed in our investigation and the packages/libraries used within those languages.

R v4.1.2	Python v3.5.6
DescTools	argparse
boot	os
caret	pandas
cowplot	numpy
data.table	matplotlib.pyplot
dplyr	pdb
effectsize	recalib
forestplot	Lifelines
ggplot2	
ggsignif	
glmnet	
lemon	
performance	
rms	
speedglm	
stringr	
survival	
viridis	

Supplementary Table 18: Dictionary connecting abbreviations used in the text to their full meaning.

Abbreviations	Meaning
ARIC	Atherosclerosis Risk in the Community
ASCVD	Atherosclerotic Cardiovascular Diseases
BIR	Borderline/ Intermediate Risk
CAD	Coronary Artery Disease
CHD	Coronary Heart Disease
GWAS	Genome Wide Association Study
MESA	Multi-Ethnic Study of Atherosclerosis
NRI	Net Reclassification Improvement
ORxSD	Odds Ratio by Standard Deviation
PCE	Pooled Cohort Equations
PRS	Polygenic Risk Score(s)
UKB or UKBB	United Kingdom Biobank
