# Supplementary material for: Ancestry-specific polygenic risk scores are risk enhancers for clinical cardiovascular disease assessments
Source: Nat Commun. 2023 Nov 4;14:7105. doi: 10.1038/s41467-023-42897-w (PMC10625612; doi:10.1038/s41467-023-42897-w)
Supplement: Supplementary file 3 — Reporting Summary [file 41467_2023_42897_MOESM3_ESM.pdf]

nature portfolio

Corresponding author(s): George Busby, Giordano Botta

Last updated by author(s): Oct 11, 2023

Reporting Summary

Nature Portfolio wishes to improve the reproducibility of the work that we publish. This form provides structure for consistency and transparency in reporting. For further information on Nature Portfolio policies, see our Editorial Policies and the Editorial Policy Checklist.

Statistics

For all statistical analyses, confirm that the following items are present in the figure legend, table legend, main text, or Methods section.

n/a	Confirmed	
☐	☒	The exact sample size (*n*) for each experimental group/condition, given as a discrete number and unit of measurement
☐	☒	A statement on whether measurements were taken from distinct samples or whether the same sample was measured repeatedly
☐	☒	The statistical test(s) used AND whether they are one- or two-sided<br>*Only common tests should be described solely by name; describe more complex techniques in the Methods section.*
☐	☒	A description of all covariates tested
☐	☒	A description of any assumptions or corrections, such as tests of normality and adjustment for multiple comparisons
☐	☒	A full description of the statistical parameters including central tendency (e.g. means) or other basic estimates (e.g. regression coefficient) AND variation (e.g. standard deviation) or associated estimates of uncertainty (e.g. confidence intervals)
☐	☒	For null hypothesis testing, the test statistic (e.g. *F*, *t*, *r*) with confidence intervals, effect sizes, degrees of freedom and *P* value noted<br>*Give P values as exact values whenever suitable.*
☒	☐	For Bayesian analysis, information on the choice of priors and Markov chain Monte Carlo settings
☒	☐	For hierarchical and complex designs, identification of the appropriate level for tests and full reporting of outcomes
☒	☐	Estimates of effect sizes (e.g. Cohen's *d*, Pearson's *r*), indicating how they were calculated

Our web collection on statistics for biologists contains articles on many of the points above.

Software and code

Policy information about availability of computer code

Data collection	No software was used for data collection
Data analysis	A complete list of functions used to analyze data using the R programming language (v4.1.2) and Python (v3.5.6) for this study appears in Supplementary Table 17

For manuscripts utilizing custom algorithms or software that are central to the research but not yet described in published literature, software must be made available to editors and reviewers. We strongly encourage code deposition in a community repository (e.g. GitHub). See the Nature Portfolio guidelines for submitting code & software for further information.

Data

Policy information about availability of data

All manuscripts must include a data availability statement. This statement should provide the following information, where applicable:

- Accession codes, unique identifiers, or web links for publicly available datasets
- A description of any restrictions on data availability
- For clinical datasets or third party data, please ensure that the statement adheres to our policy

The UK Biobank is available to qualified researchers through https://www.ukbiobank.ac.uk/enable-your-research/apply-for-access. Both the MESA and ARIC datasets are freely available through dbGaP with accession codes phs000209.v2.p1 and phs000280.v3.p1, respectively. The 1000 Genomes dataset is freely available from multiple sources, including https://www.internationalgenome.org/. The PRS-CSx and Finemapping tools, along with all relevant data, were accessed

through GitHub. The polygenic risk scores we developed are available for non-commercial use upon request. UK Biobank data was accessed under application ID 40692.

Research involving human participants, their data, or biological material

Policy information about studies with human participants or human data. See also policy information about sex, gender (identity/presentation), and sexual orientation and race, ethnicity and racism.

Reporting on sex and gender	We use the term sex and categories male and female throughout to classify individuals into groups. Sex here refers to sex at birth and is self-reported in the datasets that we used.
Reporting on race, ethnicity, or other socially relevant groupings	We use the term genetic ancestry throughout to group individuals based on genetic similarity. In the Introduction, we qualify our definition: "Throughout, we use the term genetic ancestry and ancestry interchangeably to refer to groupings of individuals who are similar to each other genetically and label these groups using current-day geographical names that relate to the continents on which most individuals belonging to that group currently reside. These labels are intended to assign individuals to groups based on their genetics alone and are not social or ethnicity identifiers. We acknowledge that these labels are imperfect, both because modern human populations rarely contain ancestry from a single region [18], but also because they enforce the artificial discretisation of continuous human genetic diversity [19]. Moreover, when grouping individuals into continental-level ancestries, as we do here, a large amount of within-group diversity is concealed. Nevertheless, because genetic variation is shared amongst individuals with similar genetic ancestry, these approximations help to assess genetic effects in groups of similar individuals, and are a necessary tradeoff between optimizing PRSs for a diverse range of populations and having sufficient data from such groups to validate and test resulting scores."
Population characteristics	In addition to sex and genetic ancestry we use definitions of disease status and relevant covariates for the three datasets that were published with these datasets. We describe these values in the Methods section (Datasets)
Recruitment	Recruitment is described in the Methods section
Ethics oversight	IRB approval for access of the ARIC data was provided by Advarra through protocol Pro00064511

Note that full information on the approval of the study protocol must also be provided in the manuscript.

Field-specific reporting

Please select the one below that is the best fit for your research. If you are not sure, read the appropriate sections before making your selection.

☒ Life sciences ☐ Behavioural & social sciences ☐ Ecological, evolutionary & environmental sciences

For a reference copy of the document with all sections, see nature.com/documents/nr-reporting-summary-flat.pdf

Life sciences study design

All studies must disclose on these points even when the disclosure is negative.

Sample size	Sample sizes were determined by the number of individuals available in the publicly available datasets obtained where both genotype data and matched clinical data. For PRS validation and testing analyses a minimum of 50 cases of Coronary Artery Disease were selected to comprise a group. Sample sizes are reported in Supplementary Table 2. We also describe in detail how the datasets were developed in Methods section 'PRS Validation and Testing'.
Data exclusions	No data were excluded.
Replication	We performed one round of replication (PRS Testing) using independent ancestry-specific datasets that were not used in the training and development of the PRS models.
Randomization	Randomization is not relevant because we selected individuals on the basis of their disease status and genetic ancestry. Individuals from the same cohorts were kept together to guard against cohort-specific effects leading to spurious results.
Blinding	Not relevant because we applied analyses to all applicable individuals within each cohort. The same analysis was performed across groups so blinding analysts to the contents of each group would not have an effect.

Reporting for specific materials, systems and methods

We require information from authors about some types of materials, experimental systems and methods used in many studies. Here, indicate whether each material, system or method listed is relevant to your study. If you are not sure if a list item applies to your research, read the appropriate section before selecting a response.

Materials & experimental systems

n/a	Involved in the study	
☒	☐	Antibodies
☒	☐	Eukaryotic cell lines
☒	☐	Palaeontology and archaeology
☒	☐	Animals and other organisms
☒	☐	Clinical data
☒	☐	Dual use research of concern
☒	☐	Plants

Methods

n/a	Involved in the study	
☒	☐	ChIP-seq
☒	☐	Flow cytometry
☒	☐	MRI-based neuroimaging
